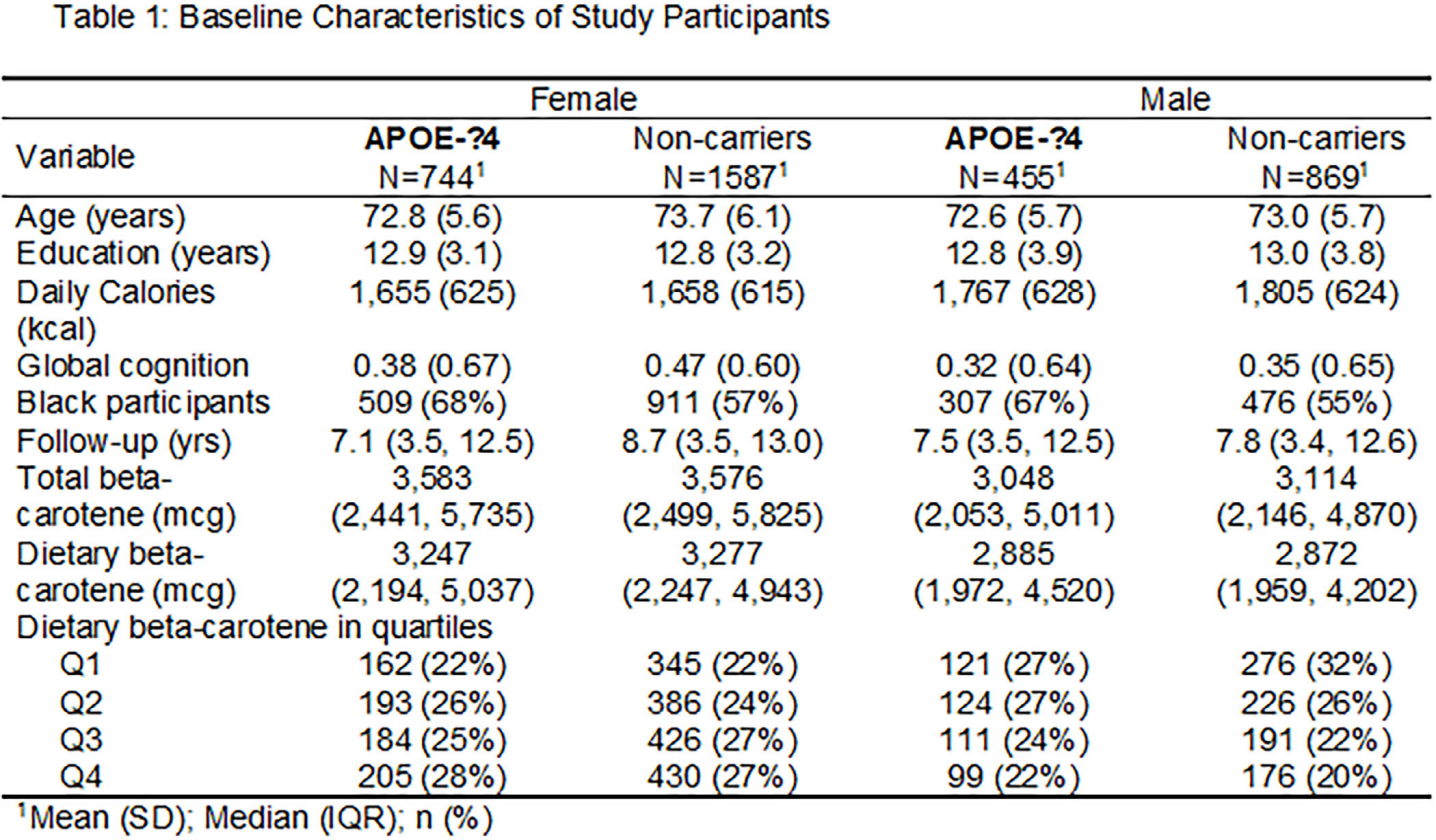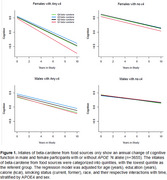# Sex‐ and APOEɛ4‐Specific Association Between Carotenoids and Cognitive Decline in Older Adults

**DOI:** 10.1002/alz70860_107192

**Published:** 2025-12-23

**Authors:** Xiaoran Liu, Todd Beck, Klodian Dhana, Pankaja Desai, Denis A Evans, Kumar B Rajan

**Affiliations:** ^1^ Rush University Medical Center, Chicago, IL, USA; ^2^ Rush Institute for Healthy Aging, Chicago, IL, USA

## Abstract

**Background:**

Carotenoids are linked to better brain health and cognitive function. However, the source of intake (dietary vs. supplemental) may differentially influence these associations. Emerging evidence suggests that dietary protective effects on cognition vary by APOEɛ4 carriership. Furthermore, sex differences in APOEɛ4‐related neurodegeneration suggest potential sex‐specific dietary effects on cognitive decline. Therefore, we aim to examine the association between dietary carotenoids and cognition, specifically by APOEɛ4 status and sex in a population‐based cohort.

**Method:**

We analyzed data from 3,655 participants in the Chicago Health and Aging Project (CHAP), a longitudinal community‐based study. Global cognition was assessed in three‐year cycles using a validated neuropsychological test battery. Dietary intake of carotenoids was assessed via a 144‐item food frequency questionnaire (FFQ), with carotenoid intake from foods only included in analyses. APOE genotyping was conducted using the hME Sequenom mass‐array platform. Carotenoid intake was analyzed both as a continuous variable and in quartiles. Linear mixed‐effects models were used to examine the associations between carotenoid intake and global cognitive decline, stratified by sex and APOEɛ4 status, adjusting for potential confounders.

**Result:**

The mean baseline age was 73.1 (SD = 5.8) years, with a mean follow‐up of 7.1 years. We observed a sex‐ and APOE‐ɛ4 specific association between dietary carotenoid intake and cognitive decline, with female APOE‐ɛ4 carriers benefiting the most. Among female APOEɛ4 carriers, higher beta‐carotene intake from food was tentatively associated with slower cognitive decline (β = 0.0030 ± 0.0016, *p* = 0.06). In quartile analyses, female APOEɛ4 carriers in the second‐highest to highest quartiles of beta‐carotene intake had significantly slower cognitive decline compared to those in the lowest quartile: β = 0.02 ± 0.011, *p* = 0.04 (2nd quartile); β = 0.03 ± 0.01, *p* = 0.014 (3rd quartile); and β = 0.02 ± 0.01, *p* = 0.05 (highest quartile). We did not observe the same association among non‐carriers or male participants.

**Conclusion:**

Higher beta‐carotene intake from food sources was associated with a slower cognitive decline in female APOE‐ɛ4 carriers but not in non‐carriers or males. These findings suggest dietary carotenoids may interact with genetic risk factors and sex‐specific mechanisms to influence cognitive aging.